# Synthesis of Oligosaccharides Derived from Lactulose (OsLu) Using Soluble and Immobilized *Aspergillus oryzae* β-Galactosidase

**DOI:** 10.3389/fbioe.2016.00021

**Published:** 2016-03-07

**Authors:** Alejandra Cardelle-Cobas, Agustin Olano, Gabriela Irazoqui, Cecilia Giacomini, Francisco Batista-Viera, Nieves Corzo, Marta Corzo-Martínez

**Affiliations:** ^1^Laboratorio de Higiene Inspección y Control de Alimentos, Departamento de Química Analítica, Nutrición y Bromatología, Universidade de Santiago de Compostela, Lugo, Spain; ^2^Departamento Bioactividad y Análisis de Alimentos, Instituto de Investigación en Ciencias de la Alimentación (CIAL, CSIC-UAM), Madrid, Spain; ^3^Departamento de Biociencias, Facultad de Química, Universidad de la República, Montevideo, Uruguay; ^4^Departamento Producción y Caracterización de Nuevos Alimentos, Instituto de Investigación en Ciencias de la Alimentación (CIAL, CSIC-UAM), Madrid, Spain

**Keywords:** oligosaccharides, lactulose, *Aspergillus oryzae*, immobilization, glutaraldehyde–agarose

## Abstract

β-Galactosidase from *Aspergillus oryzae* offers a high yield for the synthesis of oligosaccharides derived from lactulose (OsLu) by transgalactosylation. Oligosaccharides with degree of polymerization (DP) ≥ 3 have shown to possess higher *in vitro* bifidogenic effect than di- and tetrasaccharides. Thus, in this work, an optimization of reaction conditions affecting the specific selectivity of *A. oryzae* β-galactosidase for synthesis of OsLu has been carried out to enhance OsLu with DP ≥ 3 production. Assays with β-galactosidase immobilized onto a glutaraldehyde–agarose support were also carried out with the aim of making the process cost-effective and industrially viable. Optimal conditions with both soluble and immobilized enzyme for the synthesis of OsLu with DP ≥ 3 were 50 °C, pH 6.5, 450 g/L of lactulose, and 8 U/mL of enzyme, reaching yields of ca. 50% (w/v) of total OsLu and ca. 20% (w/v) of OsLu with DP 3, being 6′-galactosyl-lactulose the major one, after a short reaction time. Selective formation of disaccharides, however, was favored at 60 °C, pH 4.5, 450 g/L of lactulose and 8 U/mL of enzyme. Immobilization increased the enzymatic stability to temperature changes and allowed to reuse the enzyme. We can conclude that the use, under determined optimal conditions, of the *A. oryzae* β-galactosidase immobilized on a support of glutaraldehyde–agarose constitutes an efficient and cost-effective alternative to the use of soluble β-galactosidases for the synthesis of prebiotic OsLu mixtures.

## Introduction

β-Galactosidases (β-d-galactoside galactohydrolase E.C. 3.2.1.23) are enzymes that present several interesting applications in the food industry. Among them, they are broadly used in the production of lactose-hydrolyzed products for lactose intolerant people or lactase-deficient people (Husain, 2010). Also the prevention of lactose crystallization (Rodríguez et al., 2008) or the increase of sweetness during the preparation of ice creams, condensed milk, etc. is interesting advantages of hydrolysis of lactose.

Other uses of β-galactosidases have been focused on oligosaccharide synthesis, such as prebiotic galacto-oligosaccharides (GOS) from lactose (Cardelle-Cobas et al., 2008a; Osman et al., 2010). However, in an attempt to obtain oligosaccharides with new glycosidic structures and improved prebiotic potential, transglycosylation from carbohydrates other than lactose has been carried out. It is particularly noteworthy the transgalactosylation from lactulose (4-O-β-d-galactopyranosyl-d-fructose). Oligosaccharides derived from lactulose (OsLu) were synthesized, for the first time, by our research group via transgalactosylation using β-galactosidases from *Aspergillus aculeatus* (Cardelle-Cobas et al., 2008b), *Kluyveromyces lactis* (Martínez-Villaluenga et al., 2008), and *Aspergillus oryzae* (Cardelle-Cobas, 2009). These oligosaccharides are (galactosyl)*_*n*_* lactulose oligomers, where *n* may vary from 2 to 10. Later, OsLu were also synthesized using β-galactosidases from *A. oryzae* (Clemente et al., 2011; Anadón et al., 2013; Guerrero et al., 2013; Algieri et al., 2014); *Bacillus circulans* (Corzo-Martínez et al., 2013; Guerrero et al., 2013); *K. lactis* and *Kluyveromyces marxianus* (Padilla et al., 2015). Overall, one of the most influencing factors in the yield of prebiotic carbohydrates synthesis is the origin of enzyme. Thus, under optimal operating conditions, OsLu yields reached values of 27, 29, and 50% using β-galactosidases of *K. lactis*, *A. aculeatus*, and *A. oryzae*, respectively (Díez-Municio et al., 2014). Based on these results, *A. oryzae* β-galactosidase seems to be the best option due to its high yield for synthesis of OsLu.

Several *in vitro* and *in vivo* studies have shown that these oligosaccharides present remarkable beneficial effects, especially on the gastrointestinal system, such as bifidogenic character (Cardelle-Cobas et al., 2009, 2011, 2012; Hernández-Hernández et al., 2012), enhancement of iron absorption (Laparra et al., 2014), as well as immunomodulatory and anti-inflammatory properties (Laparra et al., 2013; Algieri et al., 2014). According to several authors (Kaneko et al., 1994; Kaplan and Hutkins, 2000; Sanz et al., 2006; Cardelle-Cobas et al., 2009), in general, oligosaccharides with degree of polymerization (DP) ≥ 3, particularly trisaccharides, show higher selectivity toward bifidobacteria. Therefore, optimization of reaction conditions affecting the specific selectivity of β-galactosidase for synthesis of OsLu with DP ≥ 3 is necessary. In this respect, the use of *A. oryzae* β-galactosidase results interesting as this enzyme offers a high specific activity of transglycosylation.

However, the use of free enzyme systems is not well accepted industrially due to the high processing time and difficulty in product recovery and enzyme reutilization (Sen et al., 2014). In this sense, immobilization of enzymes is a widely used technique that introduces a number of advantages face to the use of soluble enzyme since it may allow the application of continuous and automated processes, an accurate control of the reaction extension, the easy separation of product, the enhancement of the enzymatic thermal stability, as well as the easy recovery and reutilization of the enzyme, making the enzyme-based processes cost-effective and industrially viable (Mosbach, 1987; van Beilen and Li, 2002; Tsakiris et al., 2004; Gaur et al., 2006). Studies carried out with lactose as substrate have been focused on trying to maximize the oligosaccharide production; however, in many cases, the yield obtained for oligosaccharides can decrease up to 20–30% due to the limitations by diffusion and it is still necessary to demonstrate if immobilized biocatalyst is a better option than free enzyme for the synthesis of oligosaccharides (Sheu et al., 1998; Neri et al., 2009). Performance of *A. oryzae* β-galactosidase immobilized onto solid supports, such as amino-epoxy Sepabeads^®^, glyoxyl-agarose, chitosan, nanoparticles, magnetic polysiloxane–polyvinyl alcohol, silica and aluminosilicate adsorbents, and so on, for oligosaccharide synthesis, lactose hydrolysis, or different applications as preparation of biosensors has been thoroughly studied (Gaur et al., 2006; Irazoqui et al., 2009; Neri et al., 2009; Ansari et al., 2015a,b; Atyaksheva et al., 2015). Up to now, no significant improvement has been obtained in terms of conversion yields as compared to soluble enzyme systems. Moreover, most of this immobilization methods result expensive and difficult to scale, which limit their use at the industrial level. In fact, despite the large number of publications on enzyme immobilization on solid matrix and its applications appeared in the literature, not much of these methods have been accepted on an industrial scale (Sen et al., 2014).

Thus, the aim of the present work was the optimization of an enzymatic transgalactosylation process using *A. oryzae* β-galactosidase fixed on a suitable support that enables to obtain efficiently functional oligosaccharides from lactulose (OsLu). For that, several assays using soluble and immobilized β-galactosidase from *A. oryzae* have been carried out studying, in both cases, the influence of different factors such as the concentration of enzyme, temperature, pH, and time in the formation of OsLu. Likewise, with the purpose of evaluating the industrial viability, reusability of immobilized enzyme was also measured.

## Materials and Methods

### Materials

*Aspergillus oryzae* β-galactosidase (E. C. 3.2.1.23), lactulose, d-fructose, 6-galactobiose, and 50% glutaraldehyde were purchased from Sigma-Aldrich (Steinheim, Germany). Sepharose 4B was supplied by Pharmacia Biotechnology (Uppsala, Sweden). d-galactose, raffinose and *o*-nitrophenyl-β-d-galactopyranoside (ONPG) from Fluka (Steimheim, Germany). Fructooligosaccharides (Orafti P95) and inulin (Orafti HP) were kindly gifted by Orafti (Tienen, Belgium).

### Enzyme Immobilization onto a Glutaraldehyde–Agarose Support

Glutaraldehyde–agarose containing 90 μmol of glutaraldehyde per gram of suction-dried gel was prepared as described previously by Guisán et al. (1997). Then, following the immobilization method of Irazoqui et al. (2007), aliquots of 1 g of suction-dried glutaraldehyde–agarose gel were incubated with 10 mL of *A. oryzae* β-galactosidase solution (previously gel-filtered on a PD-10 column to remove low molecular weight molecules) containing 2 mg/mL of protein in 50 mM sodium phosphate buffer at pH 7.0 (immobilization buffer). The suspension was gently agitated at room temperature for 24 h and washed in a sintered glass filter with immobilization buffer and equilibrated with 20 mM sodium carbonate buffer at pH 10.0. The derivative was suspended in 26.4 mM sodium borohydride solution in 20 mM sodium carbonate buffer pH 10.0 at a ratio of 1 g of suction-dried gel to 14 mL of total volume. The mixture was gently stirred for 30 min at room temperature, washed with 50 mM sodium acetate buffer, pH 5.3 (activity buffer), and stored at 4 °C.

### Determination of Enzyme Activity

The β-galactosidase activity of *A. oryzae* was measured using ONPG as substrate. For the soluble form, 100 mg of β-galactosidase were dissolved in 750 μL of 0.1 M acetate buffer at pH 5.0. Enzyme solution was shaking at room temperature for 30 min until its complete dissolution and centrifuged 10 min at 10000 rpm. Afterwards, 50 μL of supernatant were added to 800 μL of ONPG solution (4.99 mM in 0.1 M sodium acetate buffer, pH 5), which was kept at 40 °C in a water bath until enzyme addition. Enzymatic activity was determined spectrophotometrically by measuring the absorbance at 410 nm of ONP (ϵ for ONP = 442 M^−1^ cm^−1^) released during reaction, by using a 1 cm path length cuvette provided with magnetic stirring. Soluble *A. oryzae* expressed a β-galactosidase activity of 9.43 units (U), where 1 unit is defined as the amount of enzyme that catalyzes the formation of 1 μmol of ONP per minute and per milliliter under the assayed conditions.

For the immobilized enzyme, activity was measured under identical conditions than for the enzyme in soluble form. Thus, 100 mg of derivative were dispersed in 750 μL of 0.1 M sodium acetate buffer pH 5. Fifty microliters of this suspension were taken and added into a cuvette containing 300 μL of ONPG and 500 μL of 0.1 M sodium acetate buffer and thermostatized at 40 °C. Absorbance measurements were recorded each 1 min, after manual shaking of the cuvette. The value for the immobilized *A. oryzae* β-galactosidase activity was 770.82 U, where 1 unit is defined, in this case, as the amount of enzyme releasing 1 μmol of ONP per minute and per gram of suction-dried gel under the above conditions.

For both soluble and immobilized forms, enzyme activity measurements were repeated five times, and the experimental error [relative standard deviation (RSD)] was lower than 3%.

### Synthesis of Oligosaccharides Derived from Lactulose (OsLu)

Batch assays for production of OsLu were carried out with soluble and immobilized preparations of *A. oryzae* β-galactosidase. The influence of process conditions, such as time (0, 1, 3, 5, 7, and 24 h), temperature (50 °C and 60 °C), pH (0.1 M acetate buffer at pH 4.5 and 0.1 M phosphate buffer at pH 6.5), and enzyme concentration (8 and16 U/mL of soluble protein or immobilized enzyme), on oligosaccharide synthesis was studied. Lactulose solutions (450 g/L) were heated before the enzyme was added and maintained at the required temperature throughout all of the experiments. Reactions were performed in individual 1.5 mL Eppendorf tubes incubated in an orbital shaker at 300 or 1000 rpm (for soluble or immobilized enzyme, respectively), using a final volume of solvent of 525 μL. Aliquots (50 μL) of sample were withdrawn from the reaction mixture at different times for 24 h and immediately immersed in boiled water for 5 min to inactivate the enzyme. Then, samples were centrifuged at 9030 × *g* for 1 min and stored at −18 °C for subsequent analysis.

Control samples were prepared in the same manner except no enzyme was added, and no changes in lactulose were detected. All assays involved the use of a fixed volume of solvent and were performed in duplicate.

### Reusability of Immobilized Enzyme

To determine the reusability of immobilized enzyme, lactulose solution (450 g/L) was incubated with the enzyme preparation (16 U/mL) in 0.1 M phosphate buffer pH 6.5 at 50 °C for 7 h. Oligosaccharides produced after each cycle were quantified by HPAEC-PAD, as described below. After each cycle of hydrolysis, the pellet containing the immobilized enzyme was recovered by filtration and reused for further cycles similarly.

### Chromatographic Characterization of OsLu Mixtures

In order to know the DP of OsLu produced after transgalactosylation process, reaction mixtures were analyzed by high-performance anion-exchange chromatography with pulsed amperometric detection (HPAEC-PAD) on an ICS2500 Dionex system (Dionex Corp., Sunnyvale, CA, USA) consisting of a GP50 gradient pump, an ED50 electrochemical detector with a gold working electrode (Ag/AgCl reference electrode). Chromatographic separations were performed following the method of Splechtna et al. (2006). Elution of carbohydrates was at room temperature on a CarboPac PA-1 column (4 × 250 mm) connected to a CarboPac PA-1 guard column (4 × 50 mm). For eluent preparation Milli Q water, 50% (w/w), NaOH (Fluka, Steinheim, Germany) and sodium acetate (NaOAc) (Panreac, Barcelona, Spain) were used. All eluents were degassed by flushing helium for 25 min. Elution of carbohydrates was in gradient of eluents A (100 mM NaOH), B (100 mM NaOH and 50 mM NaOAc), and C (100 mM NaOH and 1 M NaOAc) mixed as follows: 100% A from 0 to 20 min and from 0 to 100% B from 20 to 70 min. After each run, the column was washed for 10 min with 100% C and reequilibrated for 15 min with the starting conditions of the employed gradient. Separations were performed at room temperature and a flow rate of 1 mL/min. Detection time and voltage parameters were set according to waveform A: (E1) 0.1 V (*t*_1_) 400 ms; (E2) −2V(*t*_2_) 10 ms; (E3) 0.6 V; (E4) −0.1 (*t*_3_) 60 ms.

For chromatographic analysis, oligosaccharide mixtures and standards were diluted until appropriate concentration and filtered through 0.22 μm syringe filter (Merck Millipore, Bedford, MA, USA) before injection of 20 μL in the chromatograph using an autosampler. Acquisition and processing of data were achieved with Chromeleon software version 6.7 (Dionex Corp., Sunnyvale, CA, USA).

Quantification of lactulose and trisaccharides was performed by external calibration using a standard solution of lactulose and raffinose, respectively. The regression coefficients of the curves for each standard were always greater than 0.99.

### MALDI–TOF–MS Analysis of OsLu Mixtures

With the purpose of confirming chromatographic results, DP of synthesized OsLu was also determined by matrix-assisted laser desorption/ionization–time-of-flight mass spectrometry (MALDI–TOF–MS). Analysis were carried out on a Voyager DE-PRO mass spectrometer (Applied Biosystems, Foster City, CA, USA) equipped with a pulsed nitrogen laser (λ = 337 nm, 3 ns pulse width, and 3 Hz frequency) and a delayed extraction ion source. Ions generated by laser desorption were introduced into a time-of-flight analyzer (1.3 m flight path) with an acceleration voltage of 25 kV, 94% grid voltage, 0.025% ion guide wire voltage, and a delay time of 200 ns in the reflector positive ion mode. Mass spectra were obtained over the *m*/*z* range of 500–5000. External mass calibration was applied using the monoisotopic [M + H]^+^ values of des-Arg1 bradykinin and angiotensin I of calibration mixture 1 of the Sequazyme Peptide Mass Standards Kit (Applied Biosystems). 2,5-Dihydroxybenzoic acid (>98%, Fluka) at 10 mg/mL in water was used as the matrix. The sample was diluted 100 times in water and mixed with the matrix at a ratio of 1:4 (v/v). One microliter of this solution was spotted onto a flat stainless steel sample plate and dried in air before analysis.

## Results and Discussion

### Synthesis and Characterization of Oligosaccharides Derived from Lactulose (OsLu)

**Figure 1:**
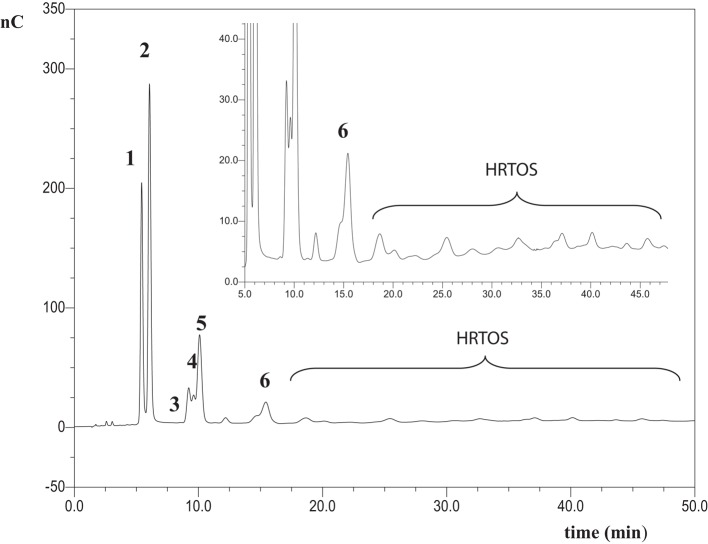
**HPAEC-PAD carbohydrate profile of oligosaccharides formed from hydrolysis of lactulose with the *A. oryzae* β-galactosidase in soluble form at pH 6.5, 50 °C, 450 g/L of lactulose and 16 U/mL of enzyme after 1 h of reaction**. Identified compounds: **1**: galactose; **2**: fructose; **3**: β-d-Gal*p*-(1 → 6)-d-Gal (6-galactobiose); **4**: β-d-Gal*p*-(1 → 6)-d-Fru (allolactulose); **5**: lactulose; **6**: β-d-Gal*p*-(1 → 6)-β-d-Gal*p*-(1 → 4)-d-Fru (6′-galactosyl-lactulose); HRTOS, high retention time oligosaccharides with DP ≥ 3.

Chromatographic profile of oligosaccharides formed after 1 h of lactulose hydrolysis with soluble β-galactosidase from *A. oryzae* is shown in Figure 1. HPAEC-PAD chromatogram obtained after 1 h of reaction with immobilized enzyme showed a similar pattern (data not shown). The use of commercial standards allowed the identification of peaks 1, 2, 3, and 5 as galactose (Gal), fructose (Fru), β-d-Gal-(1→6)-d-Gal (6-galactobiose), and lactulose, respectively. Chromatographic profile also showed the formation of allolactulose (β-d-Gal-(1→6)-d-Fru, peak 4) and 6′-galactosyl-lactulose (β-d-Gal-(1→6)-β-d-Gal-(1→4)-β-d-Fru, peak 6) being this trisaccharide the major OsLu produced. Identification of peaks 4, 6 and was done using pure standards previously obtained in our laboratory, which have been perfectly characterized by NMR and purified by semi-preparative HPLC-RI from different oligosaccharide mixtures obtained by lactulose hydrolysis and transgalactosylation with β-galactosidases from *K. lactis* and *A. aculeatus* (Cardelle-Cobas et al., 2008a,b; Martínez-Villaluenga et al., 2008). Peaks marked with an asterisk could not be identified. These might correspond to oligosaccharides with a DP ≥ 3 and have been named high retention time oligosaccharides (HRTOS). These results are supported by analysis of the reaction mixtures by MALDI–TOF, as MS spectra showed a DP of oligosaccharides comprised between 2 and 8 (Figure S1 in Supplementary Material). These results are consistent with those reported in the literature for mixtures using the same enzyme and lactose as substrate, indicating that when *A. oryzae* is used for oligosaccharide synthesis, the predominant linkages formed are β (1→6) and di- to hexa-saccharides are obtained (Toba et al., 1985; Albayrak and Yang, 2002; Martín-Huerta et al., 2010; Hernández-Hernández et al., 2011).

After characterization of OsLu mixtures, the effect of different reaction conditions, namely enzyme concentration, temperature, and pH, on the specific selectivity of *A. oryzae* β-galactosidase, in both soluble and immobilized form, for the synthesis of OsLu was studied to optimize the production of OsLu with DP ≥ 3 i.e. HRTOS.

### Optimization of the Formation of OsLu with Soluble and Immobilized β-Galactosidase

#### Effect of Enzyme Concentration

**Figure 2:**
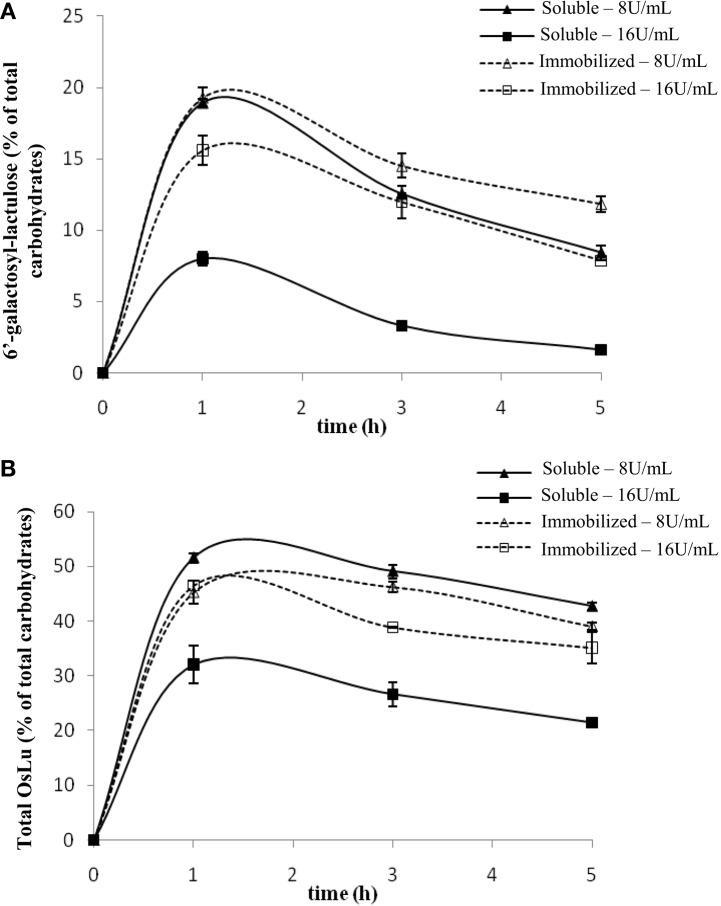
**Effect of enzyme concentration on (A) 6′-galactosyl-lactulose and (B) total OsLu production during the enzymatic hydrolysis of lactulose (450 g/L) with soluble (continuous line) and immobilized (dotted line) *A. oryzae* β-galactosidase in 0.1 M buffer sodium phosphate pH 6.5 at 50 °C: (▲, Δ) 8 U/mL; (■, □) 16 U/mL**.

The initial conditions used for the synthesis of OsLu with *A. oryzae* β-galactosidase were the optimal conditions of OsLu formation with *A. aculeatus* β-galactosidase, which were determined previously in our laboratory (Cardelle-Cobas et al., 2008b). *A. oryzae* and *A. aculeatus* belong to the same genus and their chromatographic profiles were similar, so that their behavior could also be similar. Thus, the preliminary assays (Figure 2) were carried out at 50 °C using 16 U/mL of soluble and immobilized β-galactosidase from *A. oryzae*, 450 g/L of lactulose in buffer sodium phosphate 0.1 M at pH 6.5 during 24 h of reaction. Under these conditions, the yields for total OsLu (Figure 2B) and trisaccharide 6′-galactosyl-lactulose (Figure 2A) were ca. 30 and 7.99% (w/v), respectively, after 1 h of reaction using soluble enzyme. With immobilized enzyme, yields of total OsLu (Figure 2B) and trisaccharide 6′-galactosyl-lactulose (Figure 2A) were close to 45 and 15% (w/v). Overall, after that, oligosaccharide hydrolysis was very fast, especially with the enzyme in soluble form.

With the purpose of decreasing the reaction velocity, further assays were carried out with a lower amount of enzyme (8 U/mL). In the case of the enzyme in soluble form, the yields of reaction increased up to ca. 20% (w/v) for the 6′-galactosyl-lactulose (Figure 2A) and, approximately, up to ca. 50% (w/v) for the total oligosaccharide present in the mixtures (Figure 2B). With the immobilized enzyme, the yield of 6′-galactosyl-lactulose also increased up to ca. 20% (w/v), but no variation was observed in the yield of total OsLu, suggesting that formed oligosaccharides have less access to the active site of the immobilized enzyme and, therefore, fewer possibilities to be hydrolyzed. Anyway, 8 U/mL was the concentration of β-galactosidase that gave rise to a major product yield and, therefore, it was employed to study the influence of the other parameters on the reactions of hydrolysis and transgalactosylation with β-galactosidase from *A. oryzae*.

#### Effect of Temperature

Temperature seems to affect oligosaccharide synthesis independently of substrate concentration. There are several examples of studies concluding that yield of oligosaccharide formation increases with increasing temperature (Boon et al., 2000; Hsu et al., 2007). Moreover, individual enzymes clearly show different characteristics in their response to temperature. In the present work, OsLu formation during lactulose hydrolysis at pH 6.5 with *A. oryzae* β-galactosidase (8 U/mL) in soluble and immobilized forms was studied at two different temperatures, 50 °C and 60 °C. Carbohydrate content of oligosaccharide mixtures obtained after lactulose hydrolysis under these reaction conditions is shown in Table 1. In general, with both soluble and immobilized enzyme, the amount of released monosaccharides increased with the temperature whereas the amount of lactulose decreased, especially with the immobilized enzyme. Regarding the trisaccharide 6′-galactosyl-lactulose, its formation was favored at 50 °C. Maximum content (ca. 20%, w/v) was reached after 1 h of reaction and no substantial differences were observed between values obtained with soluble and immobilized enzyme. For disaccharide formation, highest content was observed at 60 °C, reaching 6-galactobiose values of ca. 7.4% (w/v) and ca. 9.5% (w/v) after 5 h with the soluble and immobilized enzyme, respectively, and allolactulose values of ca. 19% (w/v) after 1 h with the soluble enzyme. Under these conditions (60 °C, 1 h) with immobilized enzyme, allolactulose could not be quantified in the reaction mixture as it coeluted along with lactulose, whose content in the reaction mixture respecting that of allolactulose was substantially higher.

**Table 1:** **Effect of temperature on the carbohydrate content present in the reactions mixtures during the hydrolysis of lactulose (450 g/L in 0.1 M buffer sodium phosphate at pH 6.5) using *A. oryzae* β-galactosidase in soluble form and immobilized (8 U/mL) on a support of glutaraldehyde–agarose**.

Enzyme	T (°C)	t (h)	Carbohydrate content (% of total carbohydrates)							Total OsLu content (%)^e^
			Galactose	Fructose	Lactulose	Gal-Gal^a^	AlloLu^b^	6′-Gal-Lu^c^	HRTOS^d^	
Soluble	50	1	6.47 ± 0.03	20.93 ± 0.99	21.03 ± 0.10	3.04 ± 0.19	1.09 ± 0.09	18.94 ± 0.27	28.49 ± 0.51	51.56 ± 0.86
		3	11.09 ± 0.21	25.69 ± 0.14	14.14 ± 0.90	5.47 ± 0.09	2.74 ± 0.50	12.54 ± 0.08	28.32 ± 0.17	49.07 ± 1.24
		5	14.64 ± 0.01	30.46 ± 0.29	12.19 ± 0.33	6.56 ± 0.02	3.79 ± 1.01	8.43 ± 0.51	23.92 ± 0.66	42.70 ± 0.63
	60	1	17.17 ± 0.25	27.20 ± 0.89	18.16 ± 0.47	6.56 ± 0.79	18.89 ± 5.93	4.83 ± 0.11	21.94 ± 0.13	52.22 ± 4.58
		3	13.91 ± 0.25	25.48 ± 0.70	11.23 ± 0.20	6.70 ± 0.10	14.78 ± 0.29	10.44 ± 0.10	28.07 ± 0.15	59.99 ± 0.41
		5	16.38 ± 0.02	25.54 ± 0.08	7.69 ± 0.16	7.37 ± 0.08	17.25 ± 0.70	6.7 ± 0.23	31.09 ± 0.07	62.41 ± 0.38
Immobilized	50	1	7.00 ± 0.40	18.00 ± 0.50	31.30 ± 1.20	5.70 ± 0.60	n.q.^f^	19.80 ± 0.70	18.20 ± 0.17	43.70 ± 2.20
		3	10.30 ± 1.40	22.20 ± 0.90	22.00 ± 3.20	7.20 ± 0.20	5.50 ± 0.20	15.80 ± 1.80	22.60 ± 0.31	51.10 ± 0.90
		5	12.60 ± 2.60	24.00 ± 1.50	16.30 ± 4.70	7.50 ± 0.10	6.00 ± 0.10	11.70 ± 1.90	27.90 ± 0.72	53.10 ± 0.70
	60	1	9.20 ± 2.00	19.10 ± 0.40	28.20 ± 0.40	4.70 ± 0.30	n.q.	18.00 ± 2.80	21.80 ± 0.47	44.50 ± 1.90
		3	18.50 ± 0.50	29.60 ± 1.50	11.40 ± 3.20	8.20 ± 0.20	n.q.	9.50 ± 1.90	22.80 ± 0.25	40.50 ± 0.70
		5	23.60 ± 0.80	33.00 ± 1.20	10.00 ± 4.70	9.50 ± 0.10	n.q.	5.00 ± 1.20	24.50 ± 0.37	39.00 ± 0.80

*^a^6-Galactobiose*.

*^b^Allolactulose*.

*^c^6′-Galactosyl lactulose*.

*^d^High retention time oligosaccharides*.

*^e^Total content in oligosaccharides derived from lactulose = Gal-Gal + AlloLu + 6′-Gal-Lu + HRTOS*.

*^f^n.q., not quantified*.

Thus, the higher formation of disaccharides together with the higher release of Gal and Fru at 60 °C indicates that oligosaccharide degradation is favored at this temperature.

Content in total OsLu was maximum at 60 °C with a value of ca. 62.4% (w/v) after 5 h of reaction with the enzyme in soluble form; however, disaccharide content constituted ca. 40% of this value. At 50 °C, the content in total OsLu, which was reached after 1 h of reaction, was lower than that at 60 °C (ca. 52%, w/v), but there was approximately only a 4% (w/v) of disaccharides and the remaining 48% was oligosaccharides with a DP ≥ 3. Similarly, with the immobilized enzyme, the highest content in total OsLu (ca. 53%, w/v) and HRTOS (OsLu with DP ≥ 3) (ca. 40%, w/v) was reached at 50 °C after 5 h of reaction. Results agree with the optimal temperature for *A. oryzae* β-galactosidase, using lactose, which is estimated around 50–55 °C (Tanaka et al., 1975; Boon et al., 2000; Tarivensen and Dogan, 2002). Based on this, temperature selected to develop the remaining experiments was 50 °C.

It should be noted that the effect of temperature on OsLu formation was less remarkable with immobilized β-galactosidase than with its soluble form. Especially, in the case of 6′-galactosyl-lactulose, the yield obtained with the immobilized enzyme is similar at the two assayed temperatures being of approximately 20% (w/v) after 1 h of reaction, while with the soluble enzyme, the difference between the two temperatures on the 6′-galactosyl-lactulose formation was about 10%.

Results indicate that the immobilization of the *A. oryza*e β-galactosidase on a support of glutaraldehyde–agarose increases enzyme stability against temperature changes.

#### Effect of pH

The pH of the reaction medium can affect the kinetics of lactulose hydrolysis and oligosaccharide synthesis, being able to selectively control the rates of OsLu synthesis and degradation and, thereby, increasing the yields. However, this may be a characteristic which varies between individual enzymes (Gosling et al., 2010).

**Figure 3:**
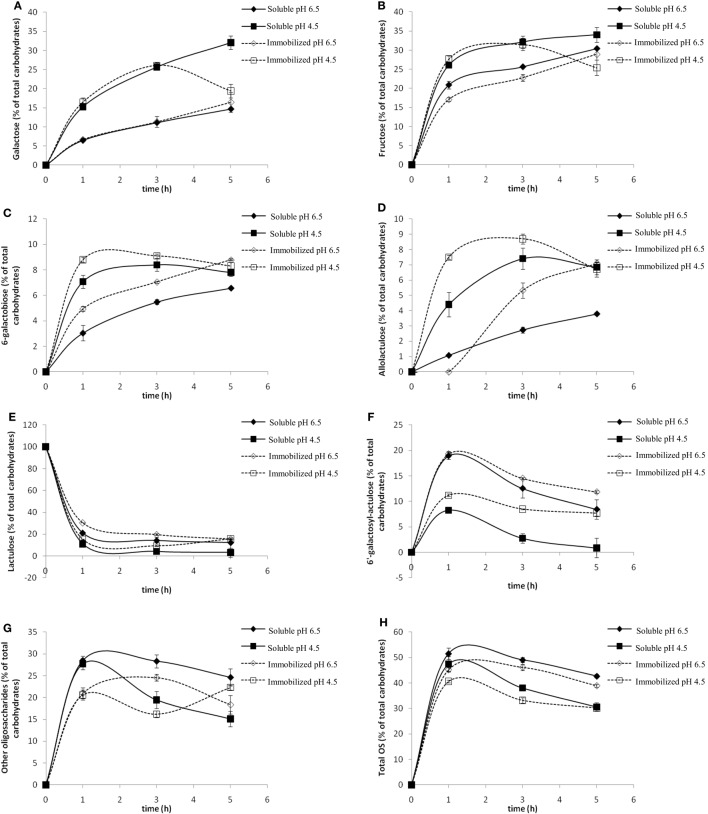
**Effect of pH on the content of (A) galactose, (B) fructose, (C) 6-galactobiose, (D) allolactulose, (E) lactulose, (F) 6′-galactosyl-lactulose, (G) other oligosaccharides (HRTOS, with DP ≥ 3), and (H) total oligosaccharides during the enzymatic hydrolysis of lactulose (450 g/L) with soluble (continuous lines, 8 U/mL) and immobilized (dotted line, 8 U/mL) *Aspergillus oryzae* β-galactosidase at 50 °C: (▲, Δ) pH 6.5; (■, □) pH 4.5**.

The effect of pH was studied at values of 4.5 and 6.5. Figure 3 shows the evolution of the content in Gal, Fru, lactulose, 6-galactobiose, allolactulose, 6′-galactosyl-lactulose, high retention time oligosaccharides (HRTOS) with DP ≥ 3, and total OsLu with the reaction time using *A. oryzae* β-galactosidase in soluble and immobilized form (8 U/mL).

At pH 6.5 using soluble and immobilized enzymes, it was observed that the percentage of released Gal was lower than at pH 4.5 (Figure 3A). The released Fru was higher than released Gal, while, at pH 4.5, no differences were found between both (Figure 3B). This suggests that Gal molecules bind to other molecules in the reaction medium, i.e., a higher degree of transgalactosylation. Moreover, the remaining lactulose amount, especially after 1 h of reaction, was notably higher than that at pH 4.5 (Figure 3E), indicating a lower degree of oligosaccharide hydrolysis. All these findings are consistent with the higher formation of 6′-galactosyl-lactulose observed at pH 6.5. The maximum formation of this trisaccharide (close to 20%, w/v) was achieved after 1 h of reaction with both soluble and immobilized enzymes at this pH, while, at pH 4.5, the maximum 6′-galactosyl-lactulose yield with soluble and immobilized enzyme was only ca. 8 and 10% (w/v), respectively (Figure 3F).

At pH 4.5, selective formation of 6-galactobiose (Figure 3C) and allolactulose (Figure 3D) was favored. Maximum yield of both disaccharides (ca. 18%, w/v) was reached after 3 h of reaction, being slightly higher with immobilized enzyme.

Regarding the formation of HRTOS (Figure 3G), no important differences were found between both pH values after 1 h of reaction. At both pH values, maximum HRTOS yield was reached after 1 h of reaction and was slightly higher with soluble enzyme (ca. 27%, w/v) than with the β-galactosidase in immobilized form (ca. 21%, w/v).

This pH-dependent behavior observed with *A. oryzae* β-galactosidase is similar to that observed with β-galactosidase from *A. aculeatus* (Cardelle-Cobas et al., 2008a,b), probably because both enzymes belong to the same genus, *Aspergillus*.

Therefore, based on results derived from all optimization assays carried out in the present work, optimal conditions for obtaining OsLu mixtures with a high content of oligosaccharides with DP ≥ 3, especially of trisaccharide 6′-galactosyl-lactulose, were 50 °C, pH 6.5, 450 g/L lactulose, and 8 U/mL of enzyme in both soluble and immobilized forms.

### Reusability of Immobilized Enzyme

One of the limitations associated with the industrial application of enzymes is their high cost and instability under operational conditions. The overall process becomes cost-effective if the preparation shows higher efficiency and reusability. As observed in Table 2, the content of lactulose increased with the cycling times. This could be attributed to the fact that lactulose was over-quantified due to co-elution with allolactulose when the former was present in high amount. Allolactulose was produced from released galactose and fructose, which is consistent with the decrease in the content of both monosaccharides. Moreover, hydrolysis of HRTOS, which is supported by the decrease observed in the content of these oligosaccharides, might also contribute to the increase observed in lactulose amount with the increase of cycling times. Regarding OsLu formation, immobilized β-galactosidase from *A. oryzae* could be used 10 times without any deterioration of its capacity to synthesize OsLu and without important decrease of OsLu yield. The RSD between assays was only 3.56%.

**Table 2:** **Effect of the multiple use of the *A. oryzae* β-galactosidase immobilized (16 U/mL) on a support of glutaraldehyde–agarose on the carbohydrate content obtained by hydrolysis of 450 g/L lactulose in buffer sodium phosphate 0.1M pH 6.5, at 50 °C after 7 h of reaction**.

Cycle no.	Carbohydrate content (% of total carbohydrates)					
	Galactose	Fructose	Lactulose	Gal-Gal^a^	6′-Gal-Lu^b^	HRTOS^c^
1	8	21.8	25.9	4.7	19.3	20.3
2	6.8	19.7	29.0	3.8	20.4	20.4
3	6.5	18.8	29.0	3.8	21.1	19.2
4	6.0	17.5	33.3	3.5	21.3	19.5
5	5.9	16.5	34.6	3.4	21.3	18.9
6	5.4	16.3	35.1	3.4	20.9	18.6
7	5.1	16.3	35.4	3.4	20.9	18.2
8	5.0	15.9	36.5	3.1	20.8	17.1
9	5.0	15.6	36.7	3.1	20.8	17.1
10	4.9	15.6	36.8	3.1	20.7	16.9

*^a^6-Galactobiose*.

*^b^6′-Galactosil lactulose*.

*^c^High retention time oligosaccharides*.

Several studies of GOS synthesis from lactose have shown that immobilization normally affects adversely the biocatalytic properties of β-galactosidase, this resulting in a GOS formation up to 20% lower than that obtained with enzyme in soluble form (Sheu et al., 1998; Neri et al., 2009). These authors established that the immobilized enzyme was probably less readily available to lactose molecules than the soluble β-galactosidase, which hampers the biocatalyst’s contact with the substrate and decreases the yield of GOS. On the contrary, results of the present work show that β-galactosidase from *A. oryzae* immobilized on a support of glutaraldehyde–agarose does not affect the transgalactosylation reaction maintaining almost unchanged the yield of OsLu.

## Conclusion

Results from optimization assays showed that *A. oryzae* β-galactosidase is selective for the production of di- or trisaccharides depending on the operating conditions, so that mixtures with different carbohydrate composition were obtained under the different studied conditions. The optimal conditions for the synthesis of 6′ galactosyl-lactulose and the formation of other oligosaccharides with DP ≥ 3, with both soluble and immobilized enzyme, were 50 °C, pH 6.5, 450 g/L of lactulose, and 8 U/mL of enzyme. Under these conditions, yields of ca. 50% (w/v) of total OsLu and ca. 20% of 6′-galactosyl-lactulose were reached after a short reaction time. Selective disaccharide formation was observed, however, at pH 4.5 and 60 °C, the maximum yield (ca. 18%, w/v) being reached after 3 h of reaction.

Immobilization increased the enzymatic stability to temperature changes and allowed to reuse the enzyme a large number of times maintaining its biocatalytic activity and, hence, the yield of OsLu.

Based on these results, we can conclude that the use, under optimal conditions, of the *A. oryzae* β-galactosidase immobilized on a support of glutaraldehyde–agarose constitutes an efficient and cost-effective alternative to the use of β-galactosidases in soluble form for the synthesis of prebiotic OsLu mixtures.

## Author Contributions

AC-C and MC-M have carried out the experimental work and the writing of a first draft of the manuscript. AO and NC have managed and supervised the work throughout all. Enzyme immobilization assays have been carried out by CG, GI and FB-V.

## Conflict of Interest Statement

The authors declare that the research was conducted in the absence of any commercial or financial relationships that could be construed as a potential conflict of interest.
