# Supplementary material for: Synthesis of Oligosaccharides Derived from Lactulose (OsLu) Using Soluble and Immobilized Aspergillus oryzae β-Galactosidase
Source: Front Bioeng Biotechnol. 2016 Mar 7;4:21. doi: 10.3389/fbioe.2016.00021 (PMC4780266; doi:10.3389/fbioe.2016.00021)
Supplement: Supplementary file 1 [file Image_1.PDF]

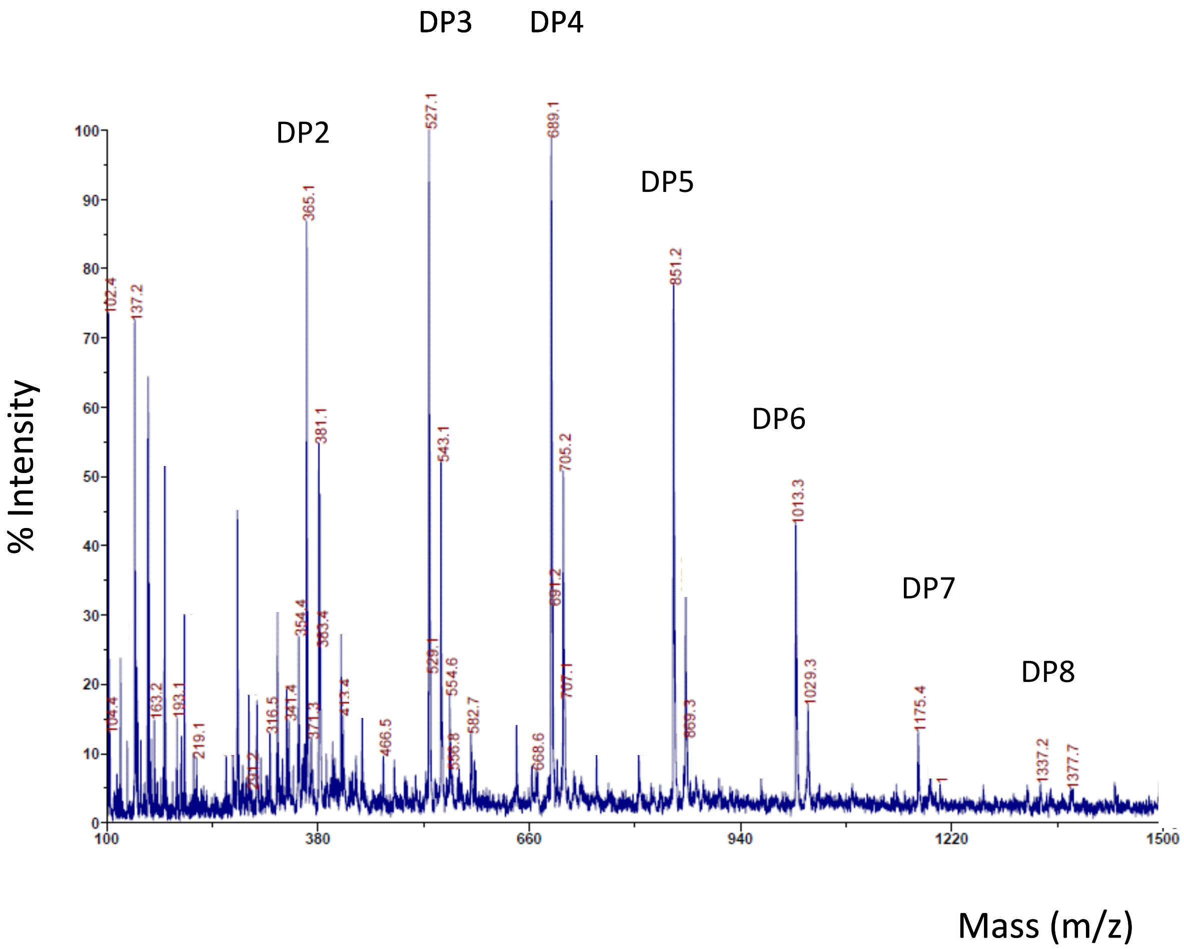

Figure S1. MALDI-TOF-MS profile of the OsLu mixture formed during enzymatic transgalactosylation of lactulose with β-galactosidase from *Aspergillus oryzae*. DP: degree of polymerization.
